# Does bedside sonographic measurement of the IVC diameter correlate with central venous pressure (CVP) in the assessment of intravascular volume in children?

**DOI:** 10.1186/2036-7902-4-S1-A7

**Published:** 2012-12-18

**Authors:** Lorraine Ng, H Khine, BH Taragin, JR Avner, M Ushay, D Nunez

**Affiliations:** 1 Department of Pediatrics, Divisions of Emergency Medicine, Children’s Hospital at Montefiore /Albert Einstein College of Medicine, Bronx, NY, USA; 2 Critical Care Medicine, Children’s Hospital at Montefiore /Albert Einstein College of Medicine, Bronx, NY, USA; 3 Department of Radiology, Children’s Hospital at Montefiore /Albert Einstein College of Medicine, Bronx, NY, USA

## Background

Previous studies demonstrated that the collapsibility index (percent decrease in inferior vena cava (IVC) diameter with inspiration) of > 50% and an IVC/Aorta ratio of < 0.8 correlated with a low intravascular volume.

## Objectives

Our study sought to determine if bedside ultrasound (BUS) measurements of the IVC diameter correlate with central venous pressure (CVP) measurements as an indicator of intravascular volume status in acutely ill children.

## Patients and methods

A convenience sample of children < 21 years-old who were admitted to the pediatric critical care unit and required CVP monitoring had BUS measurements of both IVC and aortic diameters with simultaneous CVP measurement. The collapsibility index (sagittal view) and IVC/Aorta ratio (transverse view) were calculated from these measurements. A CVP ≤ 8 mmHg was considered as a marker for decreased intravascular volume.

## Results

Of the 51 participants, 21 (43%) had a CVP < 8 mmHg. Eight of 51 (16%) children had a collapsibility index > 50% and 8 of 43 (18%) had an IVC/Aorta ratio of < 0.8. The sensitivity of a collapsibility index ≥ 0.5 to predict a CVP ≤ 8 mmHg was 14%, the specificity was 83%, the positive predictive value was 38% and the negative predictive value was 57%. Neither collapsibility index (r=-0.23, p = 0.11) nor IVC/Aorta (r=-0.19, p = 0.22) correlated with CVP in assessing intravascular volume in our study population.

## Conclusions

Based on these data, the IVC and aortic measurements by BUS are not reliable indicators of intravascular volume (as determined by CVP) in acutely ill children.
